# Anomalous superficial peroneal nerve and variant cutaneous innervation of the sural nerve on the dorsum of the foot: a case report

**DOI:** 10.1186/1757-1626-2-197

**Published:** 2009-11-18

**Authors:** Somayaji Nagabhooshana, Venkata Ramana Vollala, Vincent Rodrigues, Mohandas Rao

**Affiliations:** 1 Department of Anatomy, Melaka Manipal Medical College (Manipal Campus), Manipal University, India; 2 Department of Anatomy, Asian Institute of Medicine, Science and Technology, Sungai Petani, Kedah, Malaysia

## Abstract

**Background:**

The superficial peroneal nerve is a branch of common peroneal nerve. There are reports about the variant course and distribution of this nerve. The sural nerve arises from the tibial nerve in the popliteal fossa. The variations of the above nerves described here are unique and provide significant information to surgeons dissecting lower limb.

**Case presentation:**

The present case is about some important anatomic variations observed in left lower limb of approximately 55 - year - old male cadaver. The variations were; high division of superficial peroneal nerve into medial and lateral branches, lateral branch descending in the anterior intermuscular septum in a peroneal tunnel before piercing the deep fascia and variant distribution of sural nerve on the dorsum of the foot. The probable clinical significances of the variation are discussed.

**Conclusion:**

Awareness of anatomical variations of superficial peroneal and sural nerves such as those presented here becomes important to avoid injury in real clinical situations.

## Background

The superficial peroneal nerve begins at the bifurcation of common peroneal nerve behind the head of the fibula. It is at first deep to peroneus longus, and then passes anteroinferiorly between the peroneus longus and peroneus brevis and finally between extensor digitorum longus and peronei to pierce the deep fascia in the distal third of leg, where it divides into medial and lateral branches.

The medial branch passes anterior to the ankle and divides into two dorsal digital nerves: one supplies the medial side of the great toe, and the other supplies the adjacent sides of the second and third toes. The smaller lateral branch crosses the front of the ankle traverses the dorsum of the foot laterally. It divides into dorsal digital branches that supply the contiguous sides of the third to fifth toes and the skin of the lateral aspect of the ankle. Both branches, especially the lateral, are at risk during the placement of portal incisions for arthroscopy. Superficial peroneal nerve supplies peroneus longus and brevis, and the skin of the lower part of the front of the leg, the greater part of the dorsum of the foot, and most of the dorsal surfaces of the toes [1].

The variations about the superficial peroneal nerve are the absence of either the lateral cutaneous branch or the medial cutaneous branch, which are seen as 8.6% and 0.8%, respectively. It has been shown that in the absence of the medial cutaneous branch, the saphenous nerve innervates the medial part of the foot, while in the absence of the lateral cutaneous branch, the sural nerve supplies the lateral part of the dorsum of the foot [2]. Sometimes, the superficial peroneal nerve pierces the intermuscular septum and passes from the lateral compartment into the anterior compartment, following a superficial course. In other cases, the medial and lateral branches divide before the superficial fascia is pierced [3]. The medial branch may be absent and may be replaced by the deep peroneal nerve. The nerve has been described as arising from the nerve to peroneus brevis [4]. The superficial peroneal nerve may bifurcate in the upper part of the leg and both the branches may remain in the lateral compartment [5]. Very rarely the superficial peroneal nerve may be seen in the anterior intermuscular septum [6,7].

The sural nerve arises from the tibial nerve in the popliteal fossa, and descends on the posterior surface of the gastrocnemius to enter the superficial fascia about the middle of the back of the leg. It is then joined by the peroneal communicating nerve and accompanies the small saphenous vein to the lateral side of the foot. It supplies the skin of the lateral side of the dorsum of the foot and the lateral side of the little toe [1]. The reported variations of cutaneous innervation of the dorsum of the foot by sural nerve include; it supplying lateral 1 1/2 toes, rarely 2 1/2 toes [4,8]. Sometimes the sural nerve may terminate at the lateral border of the foot without providing any digital branches [4]. The close relationship of the sural nerve with the small saphenous vein may lead to sural nerve damage (nerve tear, neuroma and scar inclusion) after small saphenous vein stripping [9,10]. The rich sensory innervation of ankle and foot is manifested through numerous communicating branches linking superficial peroneal and sural nerves on the anterolateral aspects of the foot. A study by Drizenko et al. (2004) in 55 dissections has showed 35 such communicating branches [11]. Kosinski (1926) dissected 226 feet and described 12 patterns of termination of the dorsal nerves of the foot stressing the great variability of these nerves [12].

## Case presentation

**Figure 1:**
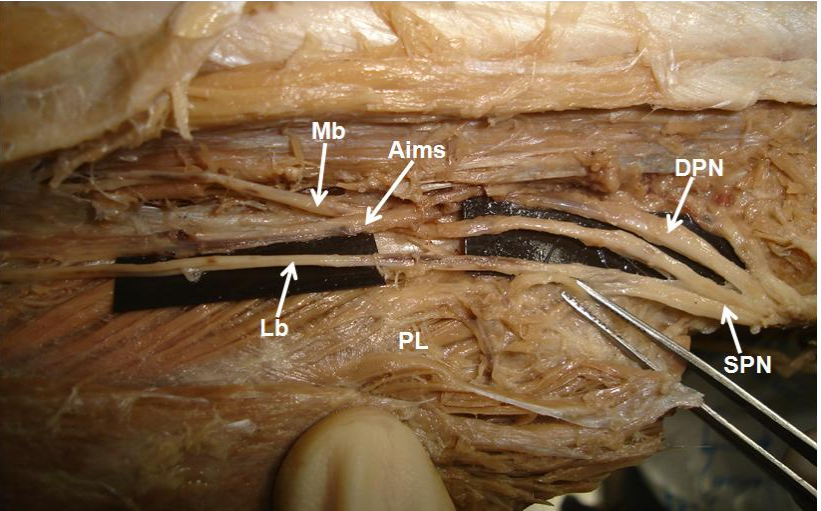
**Photograph showing high division of superficial peroneal nerve**. SPN. Superficial peroneal nerve, Mb. Medial branch, Lb. Lateral branch, Aims. Anterior intermuscular septum, DPN. Deep peroneal nerve, PL. Peroneus longus.

**Figure 2:**
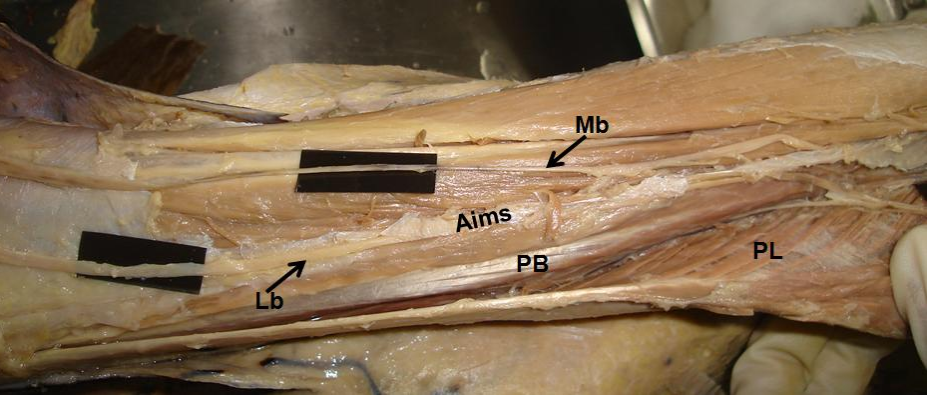
**Anterolateral aspect of leg showing branches of superficial peroneal nerve**. Mb. Medial branch, Lb. Lateral branch, Aims. Anterior intermuscular septum, PL. Peroneus longus, PB. Peroneus brevis.

**Figure 3:**
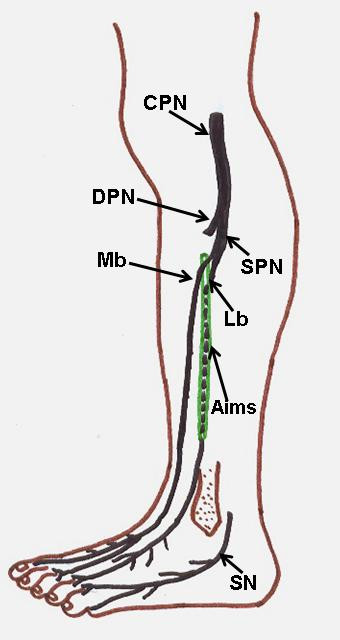
**Schematic diagram showing high division of superficial peroneal nerve, lateral branch descending in the anterior intermuscular septum and variant cutaneous innervations of dorsum of the foot**. CPN. Common peroneal nerve, DPN. Deep peroneal nerve, SPN. Superficial peroneal nerve, Mb. Medial branch, Lb. Lateral branch, Aims. Anterior intermuscular septum, SN. Sural nerve.

During routine dissection classes to medical students, we observed some important anatomic variations in left lower limb of approximately 55 - year - old male cadaver. The variations were; high division of superficial peroneal nerve into medial and lateral branches (Figure 1) and variant distribution of sural nerve on the dorsum of the foot. The medial branch arose from the superficial peroneal nerve at the proximal calf beneath the crural fascia and pierced the anterior intermuscular septum to enter the superficial fascia of anterior compartment about 23 cm proximal to the ankle (Figure 2). However, the lateral branch descended in the anterior intermuscular septum in a peroneal tunnel of 11.2 cm length, pierced the deep fascia of lateral compartment about 12 cm proximal to the ankle (Figure 3) and gave cutaneous branches to the adjacent skin of front of leg. Both medial and lateral branches were providing motor branches to peroneus longus and brevis before piercing the deep fascia.

**Figure 4:**
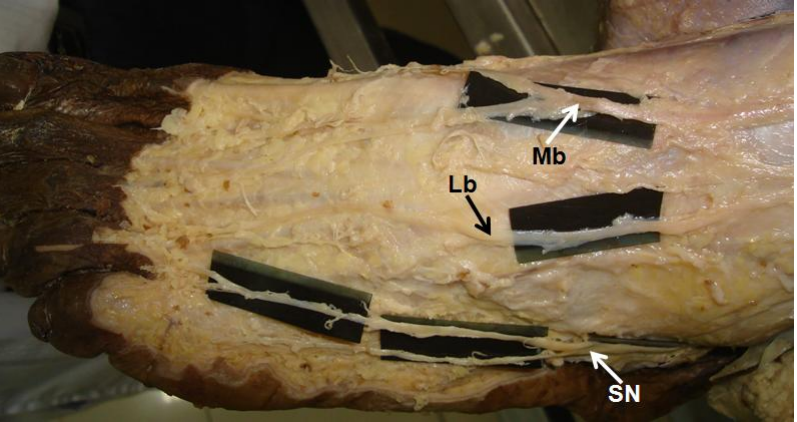
**Dissection of dorsum of foot showing variant distribution of cutaneous nerves**. Mb. Medial branch, Lb. Lateral branch, SN. Sural nerve.

On the dorsum of the foot the medial branch divided into two dorsal digital nerves; one was supplying the medial side of the great toe, and the other supplying the adjacent sides of the second and third toes. Some direct branches were seen from the medial branch supplying medial border of the foot, which is normally supplied by saphenous nerve. The lateral branch traversed the dorsum of the foot laterally. It divided into dorsal digital branches that supplied skin of the lateral aspect of the ankle and contiguous sides of the third and fourth toes. The skin over the lateral side of the foot and the sides of fourth and fifth toes were supplied by sural nerve (Figure 4). The present findings coincide with K3 variety of Kosinski's classification of termination of the dorsal nerves of the foot [12].

## Discussion

The anatomy of the superficial peroneal nerve is of critical importance to surgeons performing a variety of procedures in the middle third of the lateral leg. So, a good knowledge of the anatomical relationships and common variations of the superficial peroneal nerve is required to prevent injuries during surgical procedures.

The variations of the superficial peroneal nerve are important in fasciotomy, fasciocutaneous flaps, traumatic and atraumatic pain syndromes of the leg, regional anesthesia of the ankle, and in the determination of the surgical procedure to foot and ankle [2,13].

It has been known that the superficial peroneal nerve and its terminal branches may be iatrogenically damaged at around the knee joint, lower leg, and ankle joint, respectively [14-16]. Superficial peroneal nerve is suggested as an important and appropriate graft, particularly when multiple and long nerve grafts are needed [17]. Also, direct biopsy from the superficial peroneal nerve is used in the diagnosis of its diseases [18]. Furthermore, this nerve is under the risk of injury during deformity surgery in childhood and adolescence. All these require a well known anatomy of this nerve and its variations.

Entrapment syndrome is an uncommon but easily diagnosed clinical problem related with the superficial peroneal nerve and its branches. Superficial peroneal nerve may divide into its terminal branches before or after piercing the crural fascia, and so the nerve itself or its terminal branches may be compressed when passing through the deep fascia [13]. In such cases, several findings such as symptoms aggravated with exercise, sensory loss on the dorsum of the foot, and swelling and sensitivity on the anterolateral parts of the leg with dorsiflexion of the ankle were reported [19]. In the present case also the superficial peroneal nerve divided into terminal branches before piercing the crural fascia. Both medial and lateral branches were passing through the anterior intermuscular septum where they can be compressed. Moreover the lateral branch descended in the anterior intermuscular septum in a peroneal tunnel and pierced the deep fascia of lateral compartment about 12 cm proximal to the ankle. There are very less reports in the literature suggesting either the superficial peroneal nerve or its branches can be seen in the anterior intermuscular septum [6,7]. During the surgical procedures, if the lateral branch of superficial peroneal nerve is not identified in either anterior or lateral compartments, surgeon should look for the nerve in the anterior intermuscular septum as in the present case. One should never cut across the anterior intermuscular septum without first examining it very carefully for the presence of the superficial peroneal nerve or its branches. Inadvertent injury to the superficial peroneal nerve may create a painful neuroma or cause unwanted numbness on the dorsum of the foot. Also, when performing a release of the superficial peroneal nerve for the entrapment or when performing resection of the superficial peroneal nerve for a neuroma, one should always evaluate the septum specifically for a branch of the superficial peroneal nerve to prevent an incomplete release or resection, which would result in a therapeutic failure.

The area where the superficial peroneal nerve pierces the deep fascia and becomes superficial was reported as the junction of the middle and lower one-thirds of the leg [13]. The distance between the piercing level and the lower end of the lateral malleolus was given as 6.5-12.3 cm, which is mainly in the lower one-third [2,13]. In the present case, the piercing of medial and lateral branches were at different levels; medial being piercing the intermuscular septum at the level about 23 cm above the ankle and the lateral branch piercing the deep fascia about 12 cm above the ankle. Even though the cases wherein the superficial peroneal nerve diverges into two and then pierces the fascia are not rare globally [5,13,16], we have observed high division of superficial peroneal nerve in only 1% of cases in our laboratory. So, it's a rare occurrence in Indian population.

Percutaneous emplacement of pins or screws, such as those used for external fixing devices, or bolts to block tibial endomedullary nails can cause nerve injuries. Superficial peroneal nerve compressive syndromes have been described where the nerve pierces the superficial fascia of the leg, causing a clinical picture similar to an L5 root disease (dysesthesia on the dorsal surface of the foot and respective toes) [20]. In all the above situations, precise identification of the course of the superficial peroneal nerve is extremely important.

Superficial peroneal nerve is usually accompanied by a small artery and vein. So, it may be used as a vascularized graft in peripheral nerve surgery by orthopaedicians. Its motor branches to the peroneal muscles can be used for a neurotization of the anterior tibial muscle in patients with L4 root injuries (polio, spinal injuries, and others) [21]. On the other hand, in plastic surgery small arteries and veins accompanying the superficial peroneal nerve may be used in vascularized skin grafts [22].

The sural nerve is often used as a donor in autogenous nerve grafting in the child as well as in the adult. Biopsy material taken from the sural nerve is of great importance in the diagnosis of neural diseases. Besides, the sural nerve is the most suitable one for use as a nerve graft in surgery. There may be some difficulties to access it surgically due to various formations of the nerve [23,24].

Knowledge of potential variant anatomy of the superficial peroneal nerve and sural nerve should aid surgeons in locating the nerves as well as avoiding potentially unnecessary complications. Awareness of the unusual variant location of lateral branch of superficial peroneal nerve in the anterior intermuscular septum in the present case will enable the surgeon to find and preserve the nerve during fasciotomy, neurolysis, neuroma resection, or bony and soft tissue reconstruction.

## Conclusion

Knowledge of anatomical variations of the nerves in the lower limbs provides information to clinicians to avoid injury to them in real clinical situations. Although there are number of variations reported regarding superficial peroneal and sural nerves the case described here is unique and it will be an interesting finding to anatomists and clinicians.

## Consent

Written informed consent was obtained from the subject's relative for publication of this case report.

## Competing interests

The authors declare that they have no competing interests.

## Authors' contributions

VRV did the literature search and wrote the case report and also obtained written consent. SN, ViR and MR conceived the study and helped to draft the manuscript. All authors had gone through the final manuscript and approved it.
